# The RstAB System Impacts Virulence, Motility, Cell Morphology, Penicillin Tolerance and Production of Type II Secretion System-Dependent Factors in the Fish and Human Pathogen *Photobacterium damselae* subsp. *damselae*

**DOI:** 10.3389/fmicb.2019.00897

**Published:** 2019-04-24

**Authors:** Mateus S. Terceti, Ana Vences, Xosé M. Matanza, Alba V. Barca, Manuel Noia, Johnny Lisboa, Nuno M. S. dos Santos, Ana do Vale, Carlos R. Osorio

**Affiliations:** ^1^Departamento de Microbioloxía e Parasitoloxía, Instituto de Acuicultura, Universidade de Santiago de Compostela – USC, Santiago de Compostela, Spain; ^2^Departamento de Bioloxía Funcional, Facultade de Bioloxía-CIBUS, Universidade de Santiago de Compostela – USC, Santiago de Compostela, Spain; ^3^Fish Immunology and Vaccinology Group, IBMC-Instituto de Biologia Molecular e Celular, Universidade do Porto, Porto, Portugal; ^4^i3S – Instituto de Investigação e Inovação em Saúde, Universidade do Porto, Porto, Portugal

**Keywords:** RstAB, CarSR, *Photobacterium damselae*, damselysin, phobalysin, T2SS, TCS

## Abstract

The RstB histidine kinase of the two component system RstAB positively regulates the expression of damselysin (Dly), phobalysin P (PhlyP) and phobalysin C (PhlyC) cytotoxins in the fish and human pathogen *Photobacterium damselae* subsp. *damselae*, a marine bacterium of the family *Vibrionaceae*. However, the function of the predicted cognate response regulator RstA has not been studied so far, and the role of the RstAB system in other cell functions and phenotypes remain uninvestigated. Here, we analyzed the effect of *rstA* and *rstB* mutations in cell fitness and in diverse virulence-related features. Both *rstA* and *rstB* mutants were severely impaired in virulence for sea bream and sea bass fish. Mutants in *rstA* and *rstB* genes were impaired in hemolysis and in Dly-dependent phospholipase activity but had intact PlpV-dependent phospholipase and ColP-dependent gelatinase activities. *rstA* and *rstB* mutants grown at 0.5% NaCl exhibited impaired swimming motility, enlarged cell size and impaired ability to separate after cell division, whereas at 1% NaCl the mutants exhibited normal phenotypes. Mutation of any of the two genes also impacted tolerance to benzylpenicillin. Notably, *rstA* and *rstB* mutants showed impaired secretion of a number of type II secretion system (T2SS)-dependent proteins, which included the three major cytotoxins Dly, PhlyP and PhlyC, as well as a putative delta-endotoxin and three additional uncharacterized proteins which might constitute novel virulence factors of this pathogenic bacterium. The analysis of the T2SS-dependent secretome of *P. damselae* subsp. *damselae* also led to the identification of RstAB-independent potential virulence factors as lipoproteins, sialidases and proteases. The RstAB regulon included plasmid, chromosome I and chromosome II-encoded genes that showed a differential distribution among isolates of this subspecies. This study establishes RstAB as a major regulator of virulence and diverse cellular functions in *P. damselae* subsp. *damselae*.

## Introduction

Two-component signal transduction systems enable bacteria to sense environmental stimuli and transfer this information across the cytoplasmic membrane to the cytoplasm (Stock et al., 2000). Such systems consist of a membrane-embedded protein kinase which acts as a sensory component, and its cognate response regulator, a cytoplasmic transcriptional factor. When the sensory component of the pair is stimulated by a specific signal, it autophosphorylates a histidine residue and then transfers the phosphate group to a conserved aspartate residue of the response regulator.

*Photobacterium damselae* subsp. *damselae* (hereafter *Pdd*) is a marine bacterium pathogenic for a variety of marine animals as well as for humans, and represents an emerging threat for fish species of financial importance in marine aquaculture (Rivas et al., 2013a; Terceti et al., 2016; Osorio et al., 2018). pPHDD1 plasmid encodes two major virulence factors, the phospholipase-D damselysin (Dly) and the pore-forming toxin phobalysin P (PhlyP) (Rivas et al., 2011; Rivas et al., 2015a). Additional virulence factors are encoded within the chromosomes and include the pore-forming toxin phobalysin C (PhlyC), the phospholipase PlpV and the collagenase ColP (Rivas et al., 2013b; Vences et al., 2017). While production of PhlyC and PlpV are almost ubiquitous traits in this subspecies, only a fraction of the isolates produce the collagenase ColP (Vences et al., 2017). Mutants in the gene encoding EpsL protein, an inner membrane component of the type II secretion system (T2SS) exhibit impaired hemolysis, phospholipase, and gelatinase activities, providing evidence that the T2SS secretes Dly, PhlyP, PhlyC, PlpV and ColP enzymes (Rivas et al., 2015b; Vences et al., 2017). However, besides these cytotoxins, the secretome of *Pdd* remains largerly uncharacterized.

Recently, a functional two-component regulatory system has been reported in this pathogen which, based on its similarity to the RstAB system originally described in *Escherichia coli*, was dubbed RstAB (Terceti et al., 2017). The *Pdd* RstAB system is thus predicted to consist of the histidine kinase RstB (locus VDA_000600) and its cognate cytoplasmic response regulator RstA (locus VDA_000601). Single *rstB* mutants exhibited a strong impairment in the expression of the three hemolysins Dly, PhlyP and PhlyC as well as in virulence in a sea bass fish model. However, the role of the putative cognate response regulator RstA has not been studied to date, and nothing is known about the role of RstAB system in the regulation of cell fitness and additional virulence traits.

In the present study, we have constructed single *rstA* mutants in the pPHDD1-harboring *Pdd* strain RM-71, as well as *rstA* and *rstB* mutants in the plasmidless strain LD-07. Notably, we found that *rstA* mutation compromises virulence for fish and hemolytic activity at levels comparable to the *rstB* mutant. In addition, the RstAB system is essential for maintenance of cell shape and size and for full swimming motility under conditions of low osmolarity, and tolerance to benzylpenicillin was impaired in *rstA* and *rstB* mutants. Mutation of either *rstA* or *rstB* strongly compromised the secretion of Dly, PhyP and PhlyC as well as of a number of T2SS -dependent proteins, some of which constitute potential novel virulence factors in *P. damselae*. The RstAB regulon comprised plasmid, chromosome I and chromosome II-encoded genes that showed a notable differential distribution among isolates of this subspecies. These results demonstrate a major regulatory role of the RstAB system in the physiology and in virulence of this important marine pathogen, and open new paradigms in the study of the RstAB regulon in marine bacteria.

## Materials and Methods

### Bacterial Plasmids, Strains, and Culture Conditions

The bacterial strains and plasmids used in this study are listed in Table 1. In addition, 83 strains of *Pdd* from diverse isolation sources used in this study in the genetic screening of genes belonging to the RstAB regulon are included in Figure 7. *Pdd* cells were routinely grown at 25°C on tryptic soy agar (TSA) or broth (TSB) supplemented with 1% NaCl (TSA-1 and TSB-1, respectively) unless otherwise stated. *Escherichia coli* strains were grown at 37°C in Luria-Bertani (LB) broth or LB agar. When necessary, antibiotics were used at the following final concentrations: kanamycin (Km) at 50 μg mL^-1^, chloramphenicol (Cm) at 20 μg mL^-1^. For growth curve analysis at two NaCl concentrations (0.5 and 1%, respectively), three replicates per strain were grown in 200 μl medium in a 96 well plate inoculated 1:100 from exponentially growing precultures (OD_600_∼0.02) and analyzed using a Biotek plate reader by measuring OD_600_ at 2 h intervals.

**Table 1:** Bacterial strains and plasmids used and constructed in this study.

Strain or plasmid	Description^a^	References/Source
**Strains *P. damselae subsp. damselae***		
RM-71	Isolated from turbot; carries pPHDD1 plasmid	Fouz et al., 1992
MT151	RM-71Δ*rstB*	Terceti et al., 2017
MT319	RM-71Δ*rstA*	This study
MT157	MT151 with pRstAB (complemented mutant); Cm^R^	Terceti et al., 2017
MT245	MT319 with pRstAB (complemented mutant); Cm^R^	This study
LD-07	Isolated from gilthead seabream; does not carry pPHDD1plasmid	Vera et al., 1991
MT341	LD-07Δ*rstA*	This study
MT340	LD-07Δ*rstB*	This study
MT341C	MT341 with pRstAB (complemented mutant); Cm^R^	This study
MT340C	MT340 with pRstAB (complemented mutant); Cm^R^	This study
AR217	RM-71Δ*epsL*	Rivas et al., 2015b
AR129	RM-71Δ*hlyA_ch_*	Rivas et al., 2013b
AR133	RM-71Δ*hlyA_pl_*	Rivas et al., 2011
AR64	RM-71Δ*dly*	Rivas et al., 2011
AR158	RM-71Δ*hlyA_pl_*Δ*hlyA_ch_*	Rivas et al., 2013b
AR119	RM-71Δ*dly*Δ*hlyA_ch_*	Rivas et al., 2013b
AR78	RM-71Δ*dly*Δ*hlyA_pl_*	Rivas et al., 2011
AR89 (3Δ)	RM-71Δ*dly*Δ*hlyA_pl_*Δ*hlyA_ch_*	Rivas et al., 2013b
***E. coli***		
DH5α	Cloning strain	Laboratory stock
S17-1-λpir	RP4-2 (Km::Tn7, Tc::Mu-1) *pro*-82 *λpir recA1 endA1 thiE1 hsdR17 creC510*	Herrero et al., 1990
β-3914	F^-^ RP4-2-Tc::Mu Δ*dapA*::(*erm-pir*) *gyrA462 zei-298*::Tn*10* (Km^R^ Em^R^ Tc^R^)	Le Roux et al., 2007
**Plasmids**		
pMRB24	Cloning vector, mob, Cm^R^	Le Roux et al., 2011
pRstAB	pMRB24 with *rstAB* genes; Cm^R^	Terceti et al., 2017
pNidkan	Suicide vector derived from pCVD442: Km^R^	Mouriño et al., 2004

*^a^Cm^R^, chloramphenicol resistance; Km^R^, kanamycin resistance: Em^R^, erythromycin resistance; Tc^R^, tetracycline resistance; Amp^R^, ampicillin resistance; Δ, gene deletion.*

### Assays for Hemolysis, Phospholipase and Gelatinase Activities

Hemolysis assays on agar plates were conducted by picking a colony of each strain previously grown on TSA-1 and inoculating it on sheep blood agar plates (Oxoid) followed by growth at 25°C. For the phospholipase/lecithinase activity assay, 3 μl of overnight cultures in TSB-1 were spotted onto TSA-1 plates supplemented with 3% egg yolk extract (Oxoid), and results were evaluated after 24 h of culture at 25°C. Hydrolysis of lecithin by the phospholipase yields water-insoluble diglycerides that cause the appearance of an opaque precipitate. The gelatinase activity assay was carried out by spotting 3 μl of a TSB-1 overnight culture onto TSA-1 plates supplemented with 1% gelatin (Oxoid), and results were developed after 48 h of incubation at 25°C by covering the agar plate surface with a 12.5% (wt/vol) HgCl_2_ solution. Hydrolysis of gelatin by the gelatinase enzyme causes the appearance of a translucent halo around the bacterial colony upon addition of HgCl_2_.

### Motility Assays

Motility assays were carried out using motility agar, which consisted of TSB (0.5 or 1% NaCl) supplemented with 0.25% bacteriological agar. For this assay, 15 single colonies per assayed strain from 18-h culture agar plates were picked with a sterile plastic tip, and stabbed into the motility agar. Plates were incubated at 25°C and diameter of motility haloes was measured after 24 h cultivation. Statistical significance of differences between mean values was assessed with Student’s *t*-test; *p* ≤ 0.05 was considered to indicate statistical significance. Mann-Whitney test was used for non-parametric comparison of two mean values.

### PCR Assays

Relevant PCR primers used in this study are listed in Supplementary Table S1. PCR reactions were routinely performed with Kapa Taq DNA polymerase (Kapa). Routinely, the following thermal cycling conditions were used: 95°C for 5 min, followed by 30 cycles of 95°C for 30 s, 52.5°C for 30 s and an elongation step of 1 min at 72°C per kb.

### Allelic-Exchange Deletion Mutant Construction and Gene Complementation

Non-polar deletions of *rstA* and *rstB* genes were constructed using PCR amplification of the 5′ and 3′ fragments of each gene, which, when fused together, would result in an in-frame deletion of more than 90% of the coding sequence. The primers used are described in Supplementary Table S1. Amplification was carried out using Hi-Fidelity Kapa *Taq* (Kapa). Allelic exchange was performed using the Km^R^ suicide vector pNidKan containing the *sacB* gene, which confers sucrose sensitivity, and R6K *ori*, which requires the *pir* gene product for replication. The plasmid constructs containing the deleted alleles were transferred from *E. coli* S17-1-λ*pir* into RM-71 strain. After conjugation for 48 h on TSA plates prepared with seawater, cells were scraped off the plate and resuspended in TSB-1. Next, 100-μl aliquots of serial decimal dilutions were spread on Thiosulfate citrate bile salts sucrose (TCBS) agar supplemented with kanamycin to select for a first recombination event. Kanamycin resistant colonies were subsequently selected on TSA plates supplemented with sucrose [15% (wt/vol)] for a second recombination event. This led to the *Pdd* mutant strains described in Table 1. Deletions were confirmed by PCR and the genome region involved was sequenced to verify that the deletion was non-polar. For complementation of the mutants, *rstAB* ORFs sequence together with the respective promoter sequence was amplified by PCR using Hi-Fidelity Kapa Taq, cloned into the Cm^R^ mobilizable vector pMRB24 and mobilized from *E. coli* S17-1-λ*pir* into mutant strains MT319 (RM-71 Δ*rstA*), MT151 (RM-71 Δ*rstB*), MT341 (LD-07 Δ*rstA*) and MT340 (LD-07 Δ*rstB*).

### Fish Virulence Assays

In order to test the effect of *rstA and rstB* deletions in virulence of *Pdd* for fish, we carried out virulence assays using European sea bass (*Dicentrarchus labrax*) and gilthead sea bream (*Sparus aurata*). Groups of 10 fish (6 ± 1.2 g for sea bass; 15 ± 3 g for sea bream) per strain tested and per dose were acclimated in 100 l aquaria at 24°C for 1 week before performing the assays. Fish were inoculated intraperitoneally with 0.1 ml of bacterial suspensions of each strain in 0.85% NaCl solution at two different doses of 10^4^ CFU/fish (for sea bass) and 10^7^ CFU/fish (for sea bream). Two control groups of 10 fish each were inoculated with 0.1 ml of sterile 0.85% NaCl solution. Fish mortality was recorded daily for 10 days after inoculation. Re-isolation and identification of the bacteria from the kidney of dead fish were performed. The protocols of animal experimentation used in this study have been reviewed and approved by the Animal Ethic Committee of the Universidade de Santiago de Compostela.

### *E*-Test Assay

To determine the susceptibility to benzylpenicillin, exponentially grown cultures of *Pdd* strains were adjusted to an optical density at 600 nm (OD_600_) of 0.5 and seeded onto TSA-1 plates in the presence of *E*-test gradient antibiotic strips (bioMérieux).

### Scanning Electron Microscopy

Exponentially growing cultures of *Pdd* strains in TSB with either 0.5 or 1% NaCl were used for scanning electron microscopy observation of cell shape and size. Bacteria were fixed for 3 h at 4°C in 4% paraformaldehyde and 2% glutaraldehyde in 0.1 M phosphate buffer, pH 7.4, and postfixed for 1.5 h in 1% osmium tetroxide in the same buffer. Samples were then washed three times in dH_2_O, dehydrated using a series of graded ethyl alcohols, chemically dried using HMDS (hexamethyldisilazane) (Sigma), sputter-coated with iridium, before finally being viewed and photographed in an Ultra Plus ZEISS scanning electron microscope. Pictures were taken at different magnifications of 5,000 × and 15,000 ×. Cell width and length values were collected for 100 cells per strain tested. Box-plot diagrams were generated using the statistics program R.

### Molecular Phylogenetic Analysis

Phylogenetic relationships of the sensor and regulator proteins of the two component system RstAB of *Pdd* with homologous proteins of other bacteria were evaluated using MEGA6 (Tamura et al., 2013). Phylogenetic trees were constructed using the neighbor-joining method (Saitou and Nei, 1987), and evolutionary distances (number of residue substitutions per site) were computed using the Maximum Composite Likelihood method (Tamura et al., 2004). Numbers at the tree nodes represent bootstrap values, expressed as a percentage of 1,000 replications.

### SDS-PAGE

To identify the proteins secreted by the T2SS including those which were under the control of the two component regulatory system RstAB, we collected extracellular products (ECPs) from several replicates of liquid cultures of *Pdd* of the following strains: RM-71^wt^, Δ*rstA*, Δ*rstB*, a deletion mutant of *epsL* gene (Δ*epsL*) encoding a component of the T2SS, and different mutant combinations for the genes encoding hemolysins Dly (*dly* gene), PhlyP (*hlyA_pl_* gene) and PhlyC (*hlyA_ch_* gene). The ECPs were obtained from cultures grown in TSB-1 at 25°C to an optical density at 600 nm (OD_600_) of 1.7, corresponding to the stationary phase of growth. Bacterial suspensions were centrifuged (6000 ×*g*, 5 min, 4°C), cell pellets discarded and the culture supernatants collected and filtered through 0.22 μm-pore size filters (Schleicher & Shuell, Dassel, Germany). Proteins from 1.5 mL cell-free culture supernatants were precipitated with 10% (wt/vol) trichloroacetic acid (TCA) for 30 min on ice and recovered by centrifugation. Protein pellets were washed in 10% (wt/vol) TCA followed by a washing in acetone, and air-dried. Precipitated proteins were solubilized in SDS-sample buffer (50 mM Tris-HCl (pH 8.8), 2% SDS, 0.05% bromophenol blue, 10% glycerol, 2 mM EDTA, and 100 mM DTT) and subjected to SDS-PAGE in 10 or 12% polyacrylamide gels using the Laemmli discontinuous buffer system (Laemmli, 1970). Proteins in the gels were stained with Coomassie Brilliant Blue. For the identification of T2SS-dependent proteins, protein bands were cut from several independent polyacrylamide gels. Thus, we also made sure that the secreted protein profiles generated were reproducible.

### Proteomic Analysis

The selected protein bands were excised from the gel, reduced with DTT, and alkylated with IAA as previously described (Gomes et al., 2013), following digestion with trypsin. The resulting peptides were desalted and concentrated using reverse phase C18 tips (ZipTips, Millipore) following the manufacturer’s instructions, eluted in 60% ACN/0.1% TFA, and allowed to dry (SpeedVac, Thermo Scientific). The ressolubilized peptides were analyzed by liquid chromatography (LC) coupled to an Orbitrap Q-Exactive mass spectrometer (Thermo Scientific) using a nano spray ionization source (Easy-Spray, Thermo Scientific). Reverse phase peptide separation was performed with an Ultimate 3000 system (Thermo Scientific) fitted with a trapping cartridge (Acclaim PepMap C18 100Å, 5 mm × 300 μm i.d., 160454, Thermo Scientific) in a mobile phase of 2% ACN, 0.1% FA at 10 μL min^-1^. After 3 min of loading, the trap column was switched in-line to a PepMap RSLC C18, 3 μm, 75 μmi.d. × 15 cm EASY-Spray analytical column (ES800, Thermo Scientific). Separation was generated by mixing A: 0.1% FA, and B: 80% ACN, 0.1% FA at 300 nL min^-1^, with the following gradient: 30 min (2.5% B to 35% B), 5 min (35% B to 95% B), 5 min (hold 95% B). Data acquisition was controlled by Xcalibur 4.0 and Tune 2.8 software (Thermo Scientific). The mass spectrometer was operated in data-dependent (dd)positive acquisition mode alternating between a full scan (m/z300-2000) and subsequent HCD MS/MS of the 10 most intense peaks from full scan (normalized collision energy of 27%). ESI spray voltage was 1.9 kV. Global settings: lock masses best (m/z 445.12003), lock mass injection Full MS, chrom. peak width (FWHM) 15 s. Full scan settings: 70 k resolution (m/z200), AGC target 3e6, maximum injection time 100 ms.dd settings: minimum AGC target 1e3, intensity threshold 1e4, charge exclusión (+) unassigned, 1, 5–8, >8, peptide match preferred, exclude isotopes on, dynamic exclusion 20 s. MS2 settings: microscans 1, resolution 17.5 k (m/z 200), AGC target 1e5, maximum injection time 100 ms, isolation window 2.0 m/z, isolation offset 0.0 m/z, spectrum data type profile. The raw data was processed using Proteome Discoverer 2.2.0.388 software (Thermo Scientific) and searched against the UniProt database for the taxonomic selection *Photobacterium* (September 2017 release) and Rapid Annotations using Subsystems Technology (RAST) server (Aziz et al., 2008). The Sequest HT search engine was used to identify tryptic peptides. The ion mass tolerance was 10 ppm for precursor ions and 0.02 Da for fragment ions. Maximum allowed missing cleavage sites was set to 2. Cysteine carbamidomethylation was set as a constant modification. Methionine oxidation and N-terminal protein acetylation were defined as variable modifications. Gene nomenclature was used following that of *Pdd* type strain CIP102761 (GenBank accession number ADBS00000000.1). For those proteins absent in CIP102761, the gene nomenclature of strain RM-71 (GenBank accession number NZ_LYBT00000000.1) was used instead.

## Results

### Mutation of *rstA* or *rstB* Impairs Hemolytic Activity in *Pdd*

In a previous study, we generated a mini-Tn*10* transposon insertional library of *Pdd* RM-71, and identified a mutant that exhibited impaired hemolytic activity. Such mutant contained a disrupted VDA_000600 locus, which encodes the histidine kinase partner RstB of the two-component regulatory system RstAB (Terceti et al., 2017). The upstream locus VDA_000601 encodes a putative cognate response regulator RstA, but to date no mutants of *rstA* gene have been constructed and assayed in *Pdd*.

**FIGURE 1:**
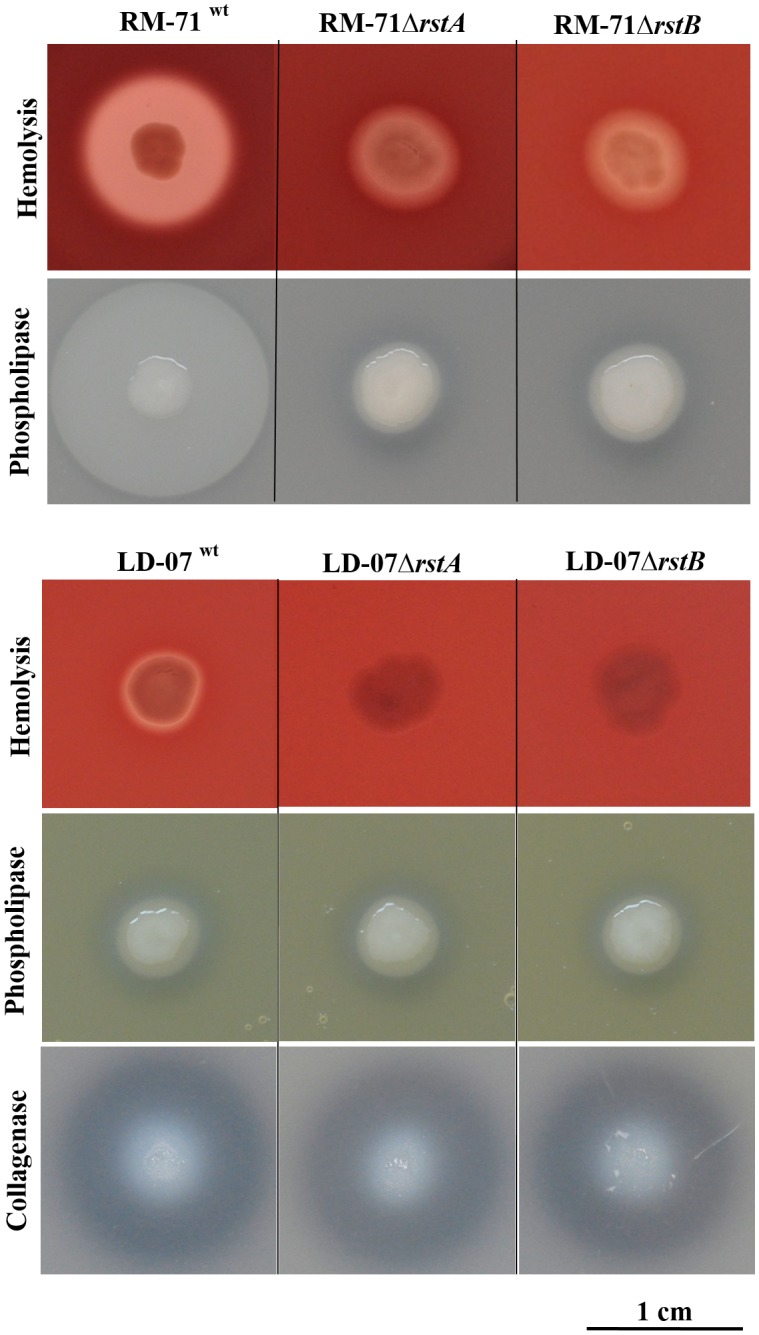
Hemolysis of sheep blood agar, lecithin degradation (phospholipase activity) and gelatin degradation (collagenase activity) phenotypes of *P. damselae* subsp. *damselae* parental strains RM-71 (top panel) and LD07 (bottom panel), and their mutant derivatives Δ*rstA* and Δ*rstB*. Scale bar, 1 cm.

Here, we constructed a *rstA* deletion mutant in RM-71 and found that its hemolytic activity for sheep blood erythrocytes was impaired at levels comparable to those of the Δ*rstB* strain (Figure 1). Hemolysis of sheep erythrocytes by RM-71, and by all the *Pdd* strains that contain the virulence plasmid pPHDD1, is due to a synergistic effect of Dly phospholipase with the two pore-forming toxins PhlyP and PhlyC (Rivas et al., 2013b). Of note, the hemolytic activity of strains that do not harbor pPHDD1 plasmid is exerted by the chromosome-encoded hemolysin PhlyC (encoded by *hlyA_ch_* gene) (Vences et al., 2017). To demonstrate the role of RstAB system in the hemolytic activity of strains lacking pPHDD1 plasmid, we selected LD-07, a plasmidless strain of *Pdd* isolated from gilthead sea bream (Table 1) that does not produce Dly and PhlyP toxins. Deletion mutants of the RstAB system were made in strain LD-07, generating the single mutants LD-07*ΔrstA* and LD-07*ΔrstB* that were seeded together with the parental strain LD-07 on sheep blood agar plates. Mutation of either *rstA* or *rstB* abolished the hemolytic activity of LD-07 against sheep erythrocytes, demonstrating that RstAB system is a positive regulator of *hlyA_ch_* gene and, therefore, is essential for hemolytic activity in plasmidless isolates (Figure 1). Complementation of the LD-07 *rstA* and *rstB* mutants with plasmid pRstAB restored the hemolytic activity against sheep erythrocytes at levels of the parental strain (data not shown).

Of the three cytotoxins, only Dly has the ability to degrade phospholipids on agar plates supplemented with lecithin, which enables the study of impairment in Dly secretion. The effect of *rstA* or *rstB* mutations in phospholipase activity in *Pdd* has not been tested to date. Here, we show that single deletions of *rstA* and *rstB* in RM-71 caused a strong impairment in the ability to degrade phospholipids (Figure 1), which demonstrates that Dly production is severely compromised in the absence of RstA or RstB. Introduction of the complementing plasmid pRstAB into the *rstA* and the *rstB* mutants of strain RM-71 restored hemolytic and phospholipase activities at the levels of the parental strain (data not shown).

### Mutations in *rstA* and *rstB* Do Not Impair PlpV-Dependent Phospholipase and ColP-Dependent Collagenase Activities

*Pdd* strains encode an ubiquitous phospholipase dubbed PlpV, whose contribution to lecithin degradation and to virulence for fish is inferior to that of Dly toxin. Plasmidless strains do not produce Dly, and yield a thin phospholipase halo in lecithin-supplemented plates caused by the minor contribution of PlpV (Vences et al., 2017). Therefore, since RM-71 produces Dly and PlpV (Vences et al., 2017), the residual precipitation halo observed in the *rstA* and *rstB* mutants in the phospholipase assay might be attributable to production of small amounts of Dly, to the contribution of PlpV, or to both. The role of the two-component system RstAB on regulation of PlpV phospholipase has not been studied so far. In order to clarify this, we seeded the parental strain LD-07 and the respective single mutants LD-07Δ*rstA* and LD-07Δ*rstB* on agar plates supplemented with egg yolk emulsion. We observed that deletion of *rstA* and *rstB* did not cause any impairment in the lecithin degradation halo, confirming that RstAB system is not a regulator of the *plpV* gene (Figure 1).

Recently, it was demonstrated that ColP collagenase has a minor contribution to virulence in LD-07 strain, and is the only gene responsible for gelatin degradation on agar plate tests (Vences et al., 2017). Since RM-71 strain lacks *colP* gene, we assayed the collagenase activity of *rstA* and *rstB* deletion mutants of strain LD-07 (*colP*-positive) on TSA-1 plates supplemented with 1% gelatin. We observed that the RstAB system is not involved in the regulation of *colP* (Figure 1).

### *rstA* and *rstB* Mutants Are Affected in Virulence for Fish

**FIGURE 2:**
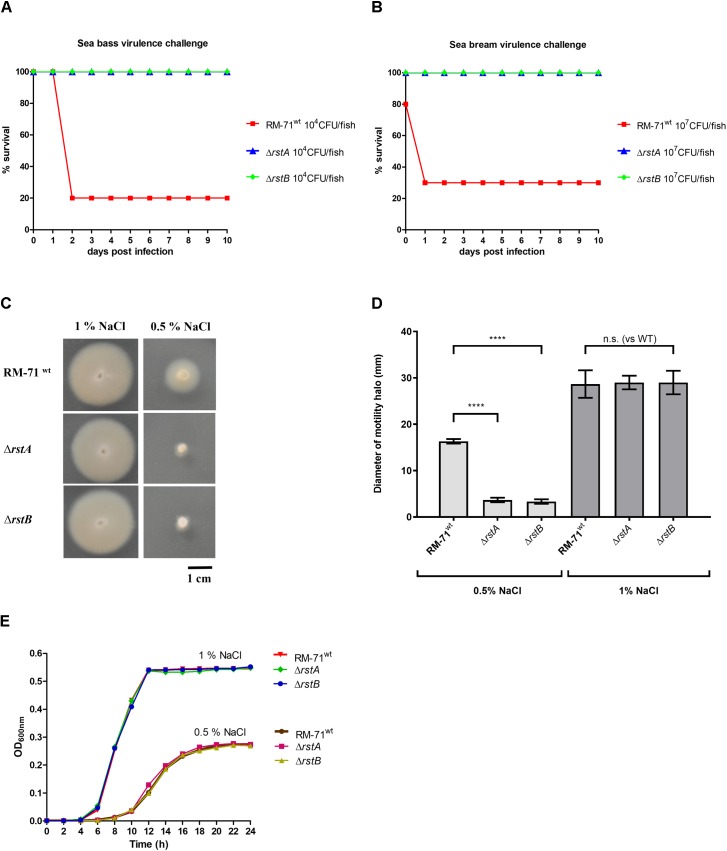
**(A,B)** Deletion of *rstA* or *rstB* genes impacts virulence for fish. Survival (%) of sea bass **(A)** and sea bream **(B)** fish after intraperitoneal injection of the *P. damselae* subsp. *damselae* parental strain (RM-71) and the Δ*rstA* and Δ*rstB* mutants. A total of 10 fish were inoculated per strain and dose assayed. The respective control fish groups (10 fish inoculated with 0.1 ml of sterile 0.85% NaCl solution) did not register any mortalities (data not shown). **(C)** Swimming motility phenotypes of parental and mutant strains in motility agar at two different NaCl concentrations. **(D)** Quantitative measurements of motility phenotypes in motility agar at two NaCl concentrations. Haloes (in mm) were measured for 15 independent colonies per strain, and mean data with standard deviation bars are shown. Four asterisks indicate a highly significant difference (*p* ≤ 0,0001) as assessed by Mann-Whitney test. **(E)** Growth curves of RM-71, Δ*rstA* and Δ*rstB* mutants in TSB with 0.5 or 1% NaCl. Mean data of three independent experiments are shown.

Previously, it was shown that *rstB* mutation drastically diminished virulence of RM-71 in a European sea bass fish model (Terceti et al., 2017). Here we wanted to test whether *rstA* mutation would compromise virulence of *Pdd* RM-71 for fish. In addition, we here extended the virulence tests to two different fish host species of this pathogen, European sea bass and gilthead sea bream. Virulence tests clearly demonstrated that the single *rstA* and *rstB* mutants are strongly impaired in their virulence for European sea bass (Figure 2A) and for gilthead sea bream (Figure 2B). All these lines of evidence suggest that RstA, the protein encoded by VDA_000601 in *Pdd*, is the cognate response regulator of the histidine kinase RstB and is necessary for hemolytic activity of *Pdd* and for virulence for fish.

### *rstAB* Mutants Show Impaired Motility and Aberrant Cell Shape and Size at 0.5% NaCl, but Not at 1% NaCl

Swimming motility is believed to constitute an important factor in the pathogenicity of *Pdd* for fish and it has been demonstrated that seawater transmits the disease (Fouz et al., 2000). Since the effect of *rstAB* genes in motility has not been assayed so far, we here investigated the behavior in motility agar of RM-71^wt^, RM-71Δ*rstA* and RM-71Δ*rstB*. We found that mutant strains were not affected in swimming motility with respect to parental strain at 1% NaCl. However, when strains were assayed for motility at 0.5% NaCl, both the *rstA* and the *rstB* mutants exhibited impaired swimming motility haloes (Figure 2C,D). Analysis of the growth curves of RM-71^wt^, RM-71Δ*rstA* and RM-71Δ*rstB* did not reveal differences in growth when the strains were exposed to the same conditions of salinity (Figure 2E). This demonstrates that the impaired motility haloes of the *rstA* and *rstB* mutants observed at 0.5% NaCl are not due to differences in growth between parental strain and mutants.

**FIGURE 3:**
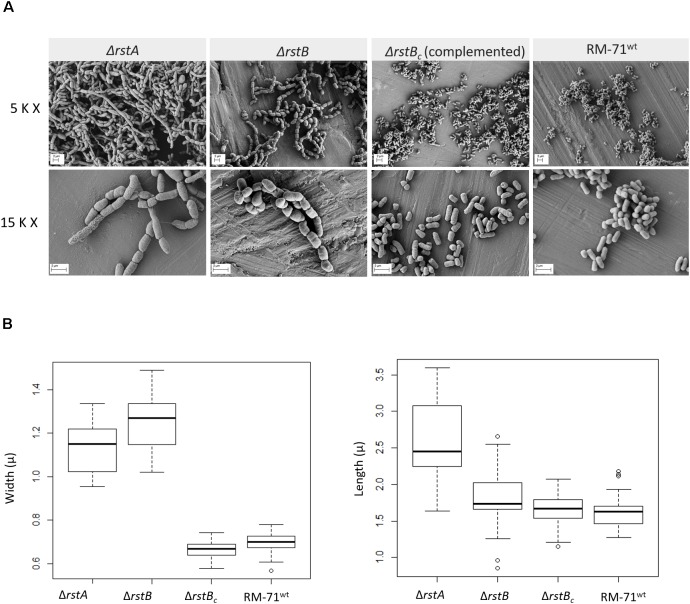
**(A)** Scanning electron microscopy of *P. damselae* subsp. *damselae* parental strain RM-71, Δ*rstA* and Δ*rstB* mutants, and complemented *rstB* mutant (MT157), grown at 25°C in TSB with 0.5% NaCl. Note the enlarged cell size of the mutants, which also form chain-like structures likely due to an impairment in daughter cell separation upon cell division. Pictures from two magnifications, 5,000 × (5 K X) and 15,000 × (15 K X) are shown, and scale bars representing 2 μm are included in each picture. **(B)** Box plot graphs showing the comparison of cell width and cell length in exponential phase cultures. Whiskers indicate min and max values.

It has been previously reported that single mutation of *rstB* does not affect cell morphology of RM-71 grown in TSB with 1% NaCl (Terceti et al., 2017). The observation that motility is impaired in *rstAB* mutants only under growth at low salinity (0.5% NaCl), prompted us to study the cell morphology of parental strain and mutants by scanning electron microscopy. We observed that, at 1% NaCl, cells of the RM-71Δ*rstA* and RM-71Δ*rstB* mutants exhibited cell shapes and sizes similar to parental strain (data not shown). However, when cells were cultured at 0.5% NaCl, differences in cell arrangement, morphology and size became evident (Figure 3). Both the *rstA* and *rstB* mutants exhibited longer and swollen cells, and most often formed chain-like structures, suggesting an impairment in daughter cell separation upon cell division. Mobilization of the complementing plasmid pRstAB into the *rstA* and *rstB* mutants caused the reversion to normal cell shapes and size (Figure 3). These results suggest that the RstAB system is essential for optimal cell division and control of cell shape and size, and for full swimming motility, under conditions of low NaCl concentrations.

### Mutation of *rstA or rstB* Decreases Tolerance to Benzylpenicillin

**FIGURE 4:**
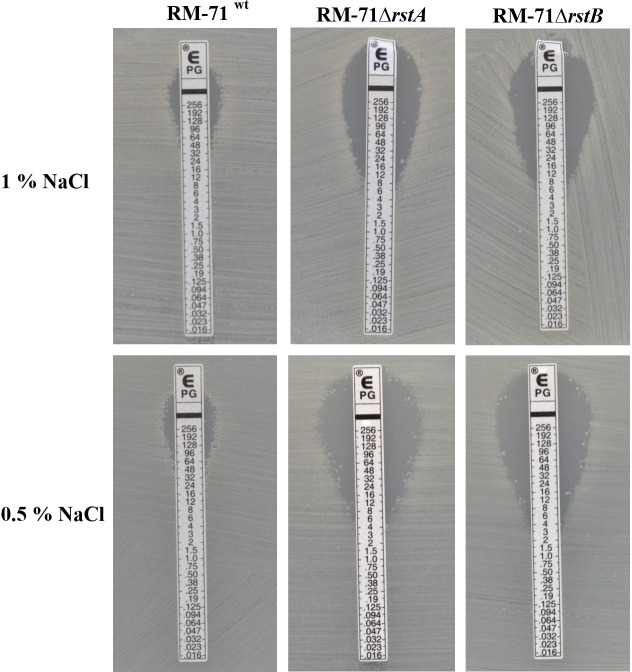
*E*-tests for benzylpenicillin, showing a decreased tolerance in both the *rstA* and the *rstB* mutants compared to parental strain RM-71.

Previous studies reported that mutations in *vprAB*/*carSR* genes (homologs of *Pdd rstAB*) cause an increased sensitivity to polymyxin B in *Vibrio cholerae* (Herrera et al., 2014; Bilecen et al., 2015). However, *rstB* mutation in *Pdd* RM-71 was not found to cause increased sensitivity to polymyxin B in a previous study (Terceti et al., 2017). As expected, we here found that the RM-71Δ*rstA* mutant was as sensitive to polymyxin as RM-71^wt^ and RM-71Δ*rstB* (data not shown). *Pdd*, as other species of the *Vibrionaceae* family, exhibit high levels of intrinsic tolerance to typically bactericidal inhibitors of cell wall synthesis, as beta lactams. A recent study reported that gene functions related to cell envelope synthesis and modification play a major role in tolerance to beta lactams by *V. cholerae* (Weaver et al., 2018). Interestingly, we found that the RM-71Δ*rstA* and RM-71Δ*rstB* mutants showed a reduction in their tolerance to benzylpenicillin in comparison to the parental strain when cultured with 1% NaCl, and this reduction was even higher when the test was conducted at 0.5% NaCl (Figure 4). These results suggest that the two-component RstAB system may be directly or indirectly involved in the regulation of the *Pdd* tolerance to beta lactams.

### *rstA* and *rstB* Mutants Are Impaired in Production of Type II Secretion System-Dependent Proteins

The four toxins Dly, PhlyP, PhlyC and PlpV are predicted to be secreted via the T2SS since *epsL* mutants were shown to be impaired in hemolytic and phospholipase activities in previous studies (Rivas et al., 2015b; Vences et al., 2017). However, the T2SS-dependent secretome of RM-71 has not been further characterized. We here wanted to gain an insight into the proteins secreted by RM-71, and into their dependence on the RstAB regulatory system. To this end, we conducted SDS-PAGE analysis of the ECPs of the parental strain and the *rstA* and *rstB* mutants. In addition, in order to evaluate the dependence of the secreted proteins on a functional T2SS, we assayed the secreted proteins of an *epsL* mutant. The *epsL* gene encodes an inner-membrane spanning protein that constitutes an essential element in the T2SS of *P. damselae* subsp. *damselae* (Rivas et al., 2015b).

**FIGURE 5:**
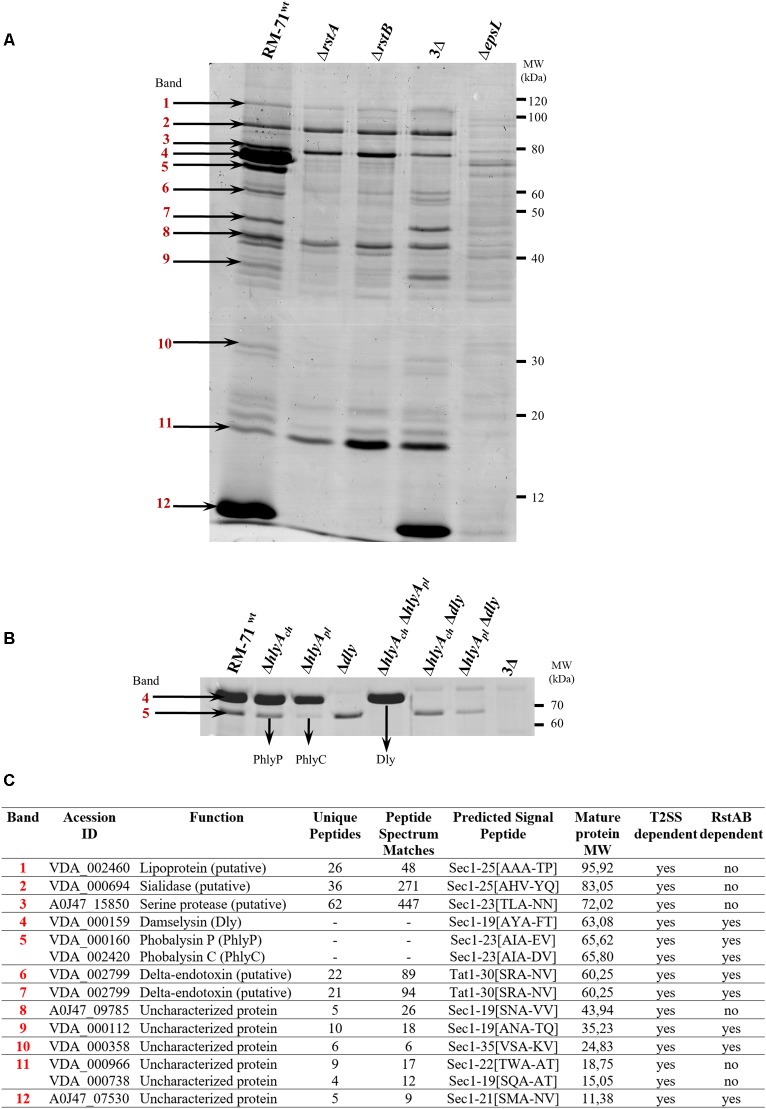
**(A)** Representative SDS-PAGE of culture supernatants from RM-71^WT^, Δ*rstA*, Δ*rstB*, 3Δ (triple mutant for *dly*, *hlyA_pl_* and *hlyA_ch_* hemolysin genes) and Δ*epsL* (defective in T2SS) strains. The identified protein bands are denoted with numbers 1 to 12. **(B)** SDS-PAGE analysis of culture supernatants from RM-71^WT^, Δ*hlyA_ch_*, Δ*hlyA_pl_*, Δ*dly*, Δ*hlyA_ch_*Δ*hlyA_pl_*, Δ*hlyA_ch_Δdly*, Δ*hlyA_pl_*Δ*dly* and 3Δ (triple mutant). Comparative analysis of the protein profiles shows that band 4 corresponds to Dly and band 5 to PhlyP+PhlyC. **(C)** Identification (gene barcodes) and characteristics of the T2SS-dependent proteins identified in this study, including those regulated by RstAB.

**FIGURE 6:**
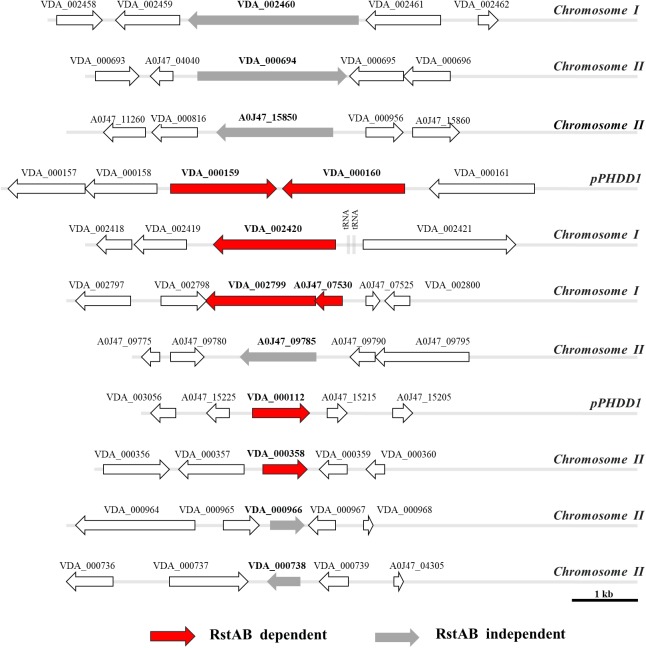
Genetic context and genomic location (chromosome I, II, or pPHDD1 plasmid) of the genes encoding the proteins identified in Figure 5.

This analysis revealed 12 protein bands whose secretion was impaired in the *epsL* mutant (hence, T2SS-dependent proteins) (Figure 5A). These bands were extracted from the gels and subjected to protein identification by mass spectrometry, and it was found that they account for 13 distinct proteins (Figure 5C). The genetic context of the genes encoding the identified proteins was analyzed and their location within the genome of *Pdd* RM-71^wt^ was elucidated (Figure 6). In addition, the proteins identified in this study were analyzed for blastP homology in other bacterial species (Supplementary Table S2) and for their domains by Pfam 31.0 (Supplementary Table S3).

The proteins related to bands 1, 2, 3, 8, and 11 are T2SS dependent but are not under control of the RstAB system, whereas the remaining bands are T2SS- and RstAB-dependent (Figure 5A). This observation indicates that mutation of the RstAB system does not prevent the correct function of the T2SS since bands 1, 2, 3, 8, and 11 are equally present in the secretome of the parental strain and in Δ*rstA* and Δ*rstB* mutants. In further support of this idea, as described above (Figure 1), *rstA* and *rstB* mutants were not affected in the lecithinase activity attributable to PlpV (mutants in the plasmidless strain LD-07), an enzyme known to be secreted via the T2SS (Vences et al., 2017).

Notably, secretion of seven T2SS-dependent proteins (included within bands 4, 5, 6, 7, 9, 10, and 12) was strongly impaired in *rstA* and *rstB* mutants, and for some of them secretion was practically abolished (Figure 5A). One of the most outstanding features in the secretome pattern of RM-71 was the presence of two major bands in the range of 60–80 kDa, that were absent in the *rstA*, *rstB* and *epsL* mutants. The molecular masses of these bands suggest that they correspond to the three hemolysins Dly, PhlyP and PhlyC, and we observed that these two major bands were absent in the protein profile of a RM-71 triple mutant for genes *dly*, *hlyA_pl_* and *hlyA_ch_* (Figure 5A). To further determine which hemolysin(s) was contained in each band, we carried out SDS-PAGE analysis of all the combinations of mutants for these three hemolysins. As a result, we corroborated that the upper intense band of ca. 75 kDa corresponded to Dly cytotoxin and the lower band of ca. 65 kDa corresponded to PhlyP plus PhlyC, with a major contribution of PhyP over PhlyC (Figure 5B). Thus, single mutation of each of the RstAB system genes, practically abolishes Dly, PhlyP and PhlyC production. In addition, as previous studies had suggested (Rivas et al., 2015b), we demonstrate that mutation of *epsL* causes a strong impairment in Dly, PhlyP, and PhlyC secretion.

Among the additional secreted proteins whose production was impaired in the *rstA* and *rstB* mutants (Figure 5C) we found potential novel virulence factors of *Pdd*. Of note, the parental strain produced high amounts of a small protein of 11 kDa (identified as A0J47_07530) whose secretion was nearly abolished in the *rstAB* and *epsL* mutants. Although the gene encoding A0J47_07530 protein is also present in the genome of the type strain CIP102761, it was not initially annotated as such in the GenBank database, although recently it has been given the locus tag VDA_RS10260. The analysis of the genetic context of A0J47_07530 unveiled that it is located upstream (and likely cotranscribed with) the gene encoding a putative delta-endotoxin (VDA_002799) (Figure 6). Indeed, this delta-endotoxin was identified as the major component of bands 6 and 7 (Figure 5A,C) and is also *rstAB* and *epsL*-dependent. MS analysis of band 6 identified delta-endotoxin peptides ranging from residues 197 to 529 whereas the delta-endotoxin corresponding to band 7 contained peptides ranging from residue 197 to 474, suggesting that band 6 corresponds to non-truncated delta-endotoxin, whereas band 7 corresponds to delta-endotoxin truncated at the C-terminus (Figure 5A).

Finally, two uncharacterized secreted proteins were also *rstAB* dependent and corresponded to the pPHDD1 plasmid-encoded VDA_000112 and the chromosome II-encoded VDA_000358. Similarity searches failed to reveal well-characterized homologs for these two proteins (Supplementary Table S2), and no conserved domains could be identified in a sequence analysis (Supplementary Table S3).

### The *P. damselae* subsp. *damselae* T2SS-Dependent Secretome Includes Additional Uncharacterized Proteins

Six additional proteins were identified as T2SS-dependent although their secretion was not affected in *rstA* and *rstB* mutants (Figure 5A,C). Since the secretome of *Pdd* has been poorly characterized so far, we judged of high interest to analyze these six proteins. VDA002460 corresponds to a T2SS-dependent putative lipoprotein, although no Pfam-A matches to known sequences were found (Supplementary Table S3). VDA_000694 corresponds to a putative sialidase and contains two Sial-lect-insert domains and a BNR_2 domain (Supplementary Tables S2, S3). It is known that pathogenic bacteria can utilize host sialic acid to form a protective coating that provides resistance to host immune response (Severi et al., 2007).

A0J47_15850 is a putative T2SS-dependent serine protease with peptidase S8 domain belonging to the family of subtilisin-like serine proteases (Bode et al., 1987). Subtilases are widespread, being found in eubacteria, archaebacteria, eukaryotes and viruses (Siezen and Leunissen, 1997). A0J47_09785 is also T2SS-dependent and corresponds to an uncharacterised *Pdd* protein with a trypsin-like domain (Supplementary Table S3) and with similarity (blastP) to proteases, metalloproteases and hemolysins (Supplementary Table S2). It also appears as Hit (19% identity) the VesB protease from *Vibrio cholerae*, which has been described as a type II-secreted protease (Gadwal et al., 2014).

VDA_000966 is also T2SS dependent and is encoded within chromosome II of *Pdd*. It has homology to hypothetical proteins and porin family proteins (Supplementary Table S2) and analysis by Pfam31.0 revealed an OMP_b-brl domain (Supplementary Table S3). Another protein identified as T2SS dependent and encoded within chromosome II is VDA_000738 which has homology with ComEA helix-hairpin-helix (HHH) repeat competence proteins of various species (Supplementary Table S2). By analysis by Pfam31.0 we have found that this protein has a HHH domain which is a short DNA-binding domain belonging to CL0198 clan superfamily (Supplementary Table S3) (Witte et al., 2008).

### Genes of the RstAB Regulon Show Differential Presence in *P. damselae* subsp. *damselae* Isolates

**FIGURE 7:**
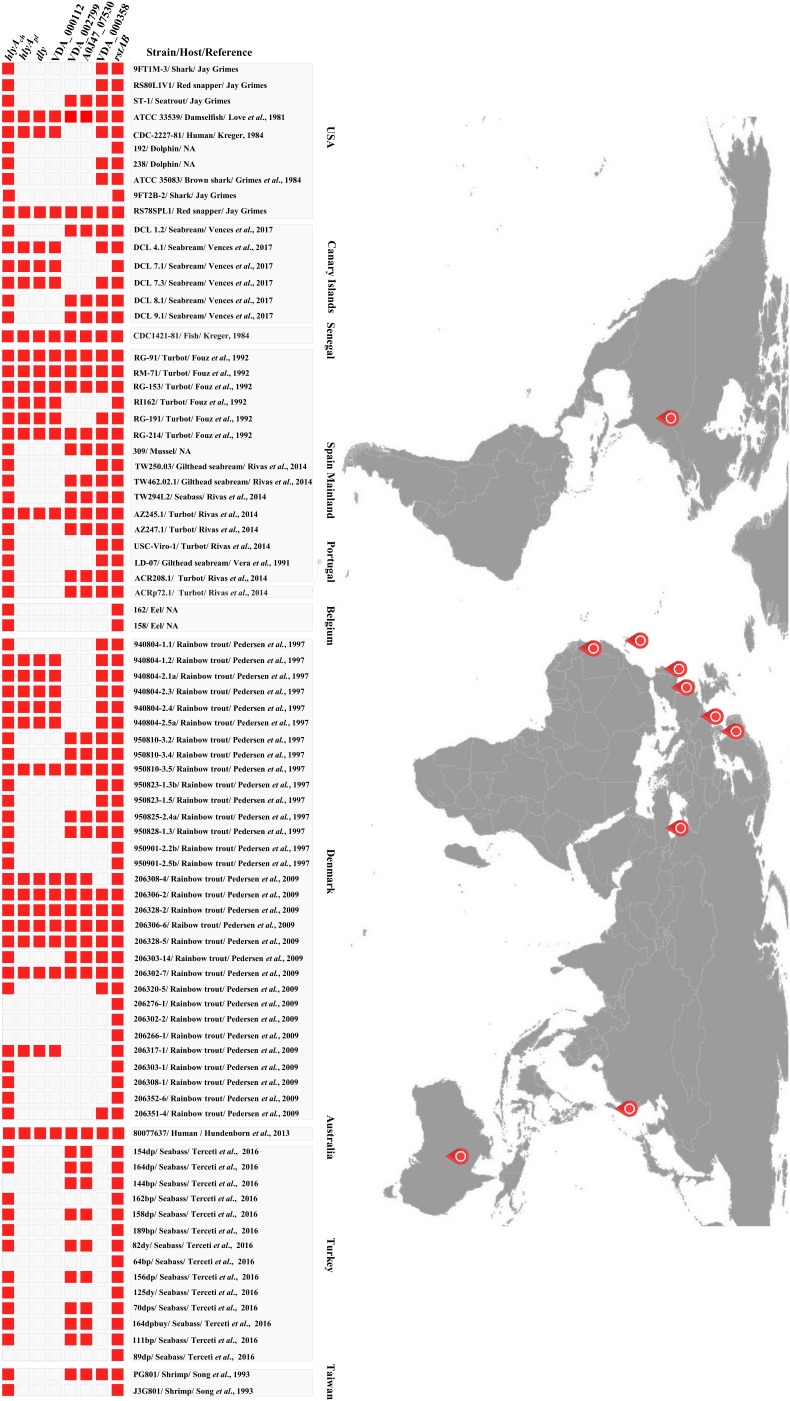
Diagramm depicting the presence of *rstAB* genes and genes of the RstAB regulon, in 83 *P. damselae* subsp. *damselae* strains isolated from different geographical locations and from different hosts including marine animals and humans.

As mentioned above, the RstAB regulon of RM-71 strain comprises at least 7 genes whose products are secreted by the T2SS. They include plasmid, chromosome I and chromosome II-encoded genes (Figure 6). In order to assess the degree of conservation of the RstAB regulon in the subspecies, a total of 83 strains of *Pdd* isolated from a variety of geographical regions and host species were tested for the presence of the following target genes: *hlyA_ch_*, *hlyA_pl_*, *dly*, VDA_000112, VDA_002799, A0J47_07530 and VDA_000358 (Figure 7). Presence of *rstAB* genes was also tested in all the strains. Some of these markers had already been tested in a fraction of these 83 isolates in previous studies (Rivas et al., 2011, 2014; Terceti et al., 2016; Terceti et al., 2018) and such previous information is also included in Figure 7.

All the genes of the RstAB regulon showed differential distribution among the isolates. The three genes *dly*, *hlyA_pl_* and VDA_000112 were present in 34% (28/83) of the isolates, and always co-occurred, an observation that is consistent with the fact that they are pPHDD1 plasmid-borne genes. The chromosome I-borne *hlyA_ch_* gene encoding PhlyC hemolysin is almost ubiquitous in the subspecies (77/83), with the exception of three Turkish and three Danish plasmidless isolates where this gene is truncated by an insertion sequence (Terceti et al., 2016, 2018). In addition, 63% (52/83) of the isolates were carriers of the gene encoding VDA_000358 (Figure 7). VDA_002799 (delta-endotoxin) and A0J47_07530 (the 11 kDa protein) always co-occurred and were present in 49% (41/83) of the assayed strains. These two genes have been reported to be inserted into a highly variable genome region that likely constitutes a hot spot for the acquisition of horizontally transferred DNA in *Pdd* (Terceti et al., 2018) (Figure 6).

Interestingly, *rstAB* genes were ubiquitous in the 83 isolates tested, demonstrating that this two-component regulatory system is highly conserved in the subspecies. Thus, the RstAB system might be used as a weak point to control outbreaks caused by both plasmid-containing and plasmidless strains. Of note, we found that homologs of the *Pdd* RstAB proteins are widely distributed in *Vibrio* and *Photobacterium* species, both pathogenic and non-pathogenic (Supplementary Figure S1).

## Discussion

Identification of the regulatory mechanisms in bacterial pathogens is of maximal interest in order to understand the environmental and host conditions that trigger the expression of virulence factors. *Pdd* is a generalist pathogen, capable of living as a free-swimming bacterium that causes outbreaks in cultured fish species when the conditions become favorable (Osorio et al., 2018). This pathogen produces a variety of toxins which are thought to be secreted through the T2SS (Rivas et al., 2015b; Vences et al., 2017). So far, the only regulatory mechanism characterized in this subspecies is the histidine kinase RstB, which is genetically linked to a putative response regulator RstA (Terceti et al., 2017).

Here we demonstrated that single mutation of *rstA* impairs virulence in sea bass and sea bream, confirming the results previously obtained with single *rstB* mutants. Studies of the role of RstAB system in fish pathogens are very scarce. Notably, mutation of *rstB* in *Edwardsiella ictaluri* caused impaired colonization and virulence in a channel catfish model (Menanteau-Ledouble and Lawrence, 2013).

*Pdd* strains are able to grow through a range of temperatures and of NaCl concentrations (Kreger, 1984; Fouz et al., 1992, 1998). Mutation of *rstA* and *rstB* genes did not affect growth in comparison to the parental strain at 25°C, either at 1% NaCl or at 0.5% NaCl. However, mutation of *rstA* or *rstB* impaired swimming motility at 0.5% NaCl. It remains to be investigated whether this reduced motility phenotype is a direct consequence of impaired flagellar function, or whether it is a consequence, at least in part, of the formation of the elongated and multicellular structures in *Pdd* grown at 0.5% NaCl. Recent studies demonstrated that salinity modulates *Pdd* swimming motility and that mutation of the chemotaxis regulator *cheA* impairs bacterial swimming (von Hoven et al., 2018). However, the connection between motility and the RstAB system in *Pdd* is so far unknown. Recent studies have shown that silencing of the *rstA* or *rstB* genes result in impaired motility, hemolysis and virulence in *Vibrio alginolyticus* (Huang et al., 2018). In *Salmonella typhimurium*, FlhA and MglB proteins involved in cell motility and chemotaxis are positively regulated by the RstAB system and a *rstB* mutant in this species showed a significant reduction in motility (Tran et al., 2016).

*rstA* and *rstB* mutants cultured at 0.5% NaCl exhibit impairment in cell separation upon cell division, as well as enlarged cell size. These mutants also exhibited decreased tolerance to benzylpenicillin. In agreement with our results, overexpression of RstA in *E. coli* made this bacterium more resistant to beta lactam antibiotics such as ampicillin (Hirakawa et al., 2003a,b). Similarly, *E. coli rstAB* mutants are hypersensitive to ketoprofen, pridinol, and troleandomycin, although the basis for these sensitivities has not been ascertained yet (Zhou et al., 2003). So far, the molecular mechanisms linking the RstAB system with tolerance to beta lactam antibiotics and with maintenance of cell size and shape in *Pdd* remain unknown. A recent study unveiled that tolerance to beta lactams by *V. cholerae* has a complex and pleiotropic nature, and that gene functions related to cell envelope synthesis and modification play a major role in such tolerance (Weaver et al., 2018). Being *Pdd* a marine bacterium, it can be anticipated that this pathogen does not encounter conditions of low salinity (0.5% NaCl) in nature. However, the abnormal phenotypes observed in the present study in *rstA* and *rstB* mutants cultured under low salinity conditions, will surely constitute the basis for further studies aimed at unveiling additional regulatory roles of RstAB systems in bacterial pathogens. Among the T2SS-dependent proteins not regulated by the RstAB system we found predicted sialidases and proteases, among others. Identifying the function of these proteins constitutes a promising challenge and will require additional research. Fish cells secrete mucus glycoproteins containing sialic acid (Kimura et al., 1994), and pathogenic bacteria can use sialidases to remove sialic acid residues from host cells and coat themselves, thus gaining resistance to components of the host’s innate immune system (Severi et al., 2007). In addition, pathogenic bacteria can bind to host sialic acid moiety to enhance adhesion and colonization. Thus, bacteria that produce sialidases might have a greater ability to colonize and stabilize themselves in the skin, gills, scales and intestines of animals that secrete mucosal glycoproteins containing sialic acids.

Mutation of *rstA* or *rstB* practically abolishes Dly, PhlyP and PhlyC production. These three hemolysins not only constitute major virulence factors for fish and mice (Rivas et al., 2011, 2013b), but are also major components of the *Pdd* secretome as revealed here in the proteome analysis. In support of this observation, a recent study on the *Pdd* transcriptome has demonstrated that the genes encoding Dly, PhlyP and PhlyC are among the most highly transcribed genes in this pathogen (Matanza and Osorio, 2018). That recent study also unveiled that the PlpV phospholipase is expressed at very low levels in comparison to the three aforementioned major hemolysins. This observation can explain why the PlpV protein band was not identified within the T2SS-dependent proteins in the present study. In addition, this study has contributed to the identification of 4 novel proteins belonging to the RstAB regulon, including plasmid-, chromosome I- and chromosome II-encoded proteins. Therefore, the response regulator RstA is predicted to recognize target genes associated with the *Pdd* chromosomes as well as genes that have been acquired by horizontal gene transfer via conjugation. Notably, our study has brought to the forefront a number of hitherto uncharacterized, T2SS-dependent and RstAB-dependent proteins, and there are very few studies on these except for the three hemolysins Dly, PhlyP and PhlyC (Kreger, 1984; Kothary and Kreger, 1985; Rivas et al., 2011, 2013b, 2015a; Vences et al., 2017). Of special interest are two genes which are likely cotranscribed as an operon, and encode the delta-endotoxin VDA_002799 and an associated 11 kDa small protein. These two genes are located within a potential hot spot for recombination within the genome of *Pdd* (Terceti et al., 2018). Delta-endotoxins, also called Cry and Cyt toxins, are pore-forming toxins predicted to cross the cytoplasmic membrane via the twin-arginine (Tat) translocation pathway (Li et al., 1991; Cygler et al., 1995; Galitsky et al., 2001). However, the role of this putative delta-endotoxin in *Pdd* is still unknown. Last, VDA_000112 and VDA_000358 are T2SS- and RstAB-dependent uncharacterized proteins encoded within plasmid pPHDD1 and chromosome II, respectively. Surely all these proteins will deserve an in-depth characterization in future studies.

*rstAB* genes are considered to be part of the PhoP/PhoQ regulon (a Mg^2+^-sensing two-component system) (Oshima et al., 2002; Ogasawara et al., 2007). While some evidence suggests that the RstAB system might respond to acidic conditions (Ogasawara et al., 2007; Huang et al., 2018), the specific stimuli that trigger the activation of the sensor histidine kinase RstB remain unknown for all the RstAB-like systems studied to date (Ogasawara et al., 2007; Campbell et al., 2007; Flamez et al., 2008; Bilecen and Yildiz, 2009; Menanteau-Ledouble and Lawrence, 2013; Tran et al., 2016; Huang et al., 2018). In contrast, a number of genes and biological functions under control of the RstAB system have been identified, demonstrating that the RstAB regulon is not predictive since it shows a high diversity among species. Functions regulated by homologous RstAB systems are as varied as pyrimidine metabolism, enterobactin biosynthesis, ferrous iron uptake and motility in *Salmonella enterica* (Tran et al., 2016), polysaccharide synthesis, biofilm formation and LPS modification in *Vibrio cholerae* (Bilecen and Yildiz, 2009; Herrera et al., 2014), or adhesion, biolfilm production, motility and hemolysis in *Vibrio alginolyticus* (Huang et al., 2018), among others.

This study has contributed to the knowledge of the bacterial RstAB regulon with a number of novel genes and functions. In addition, we here demonstrated that the RstAB regulon of *Pdd* RM-71 comprises genes of differential presence among strains, and no geographical or isolation source patterns could be identified. To summarize, the RstAB two-component system plays a major role in regulation of virulence and of many aspects of cell physiology of *Pdd*. Ongoing studies are expected to unveil the roles of the novel RstAB-regulated genes reported in this study.

## Ethics Statement

The protocols of animal experimentation used in this study have been reviewed and approved by the Animal Ethic Committee of the Universidade de Santiago de Compostela.

## Author Contributions

MT conceived the study, designed and performed the experiments, analyzed the data, and wrote the first draft of the manuscript. AV, XM, and AB performed analyses and interpreted the results. MN performed the scanning electron microscopy analyses. JL and NdS contributed to data analysis. AdV conceived the study, designed the protein analyses and data interpretation, and contributed to the writing of the manuscript. CO conceived and supervised the study, designed the experiments, interpreted the results, and wrote the manuscript. All the authors read and approved the final manuscript.

## Conflict of Interest Statement

The authors declare that the research was conducted in the absence of any commercial or financial relationships that could be construed as a potential conflict of interest.
