# Supplementary material for: The RstAB System Impacts Virulence, Motility, Cell Morphology, Penicillin Tolerance and Production of Type II Secretion System-Dependent Factors in the Fish and Human Pathogen Photobacterium damselae subsp. damselae
Source: Front Microbiol. 2019 Apr 24;10:897. doi: 10.3389/fmicb.2019.00897 (PMC6491958; doi:10.3389/fmicb.2019.00897)
Supplement: Supplementary file 1 [file Data_Sheet_1.PDF]

Supplementary Table S1. Oligonucleotides used in this study.

Target and oligonucleotides	Sequence	bp amplified	Reference
***rstA* deletion**			
1-2 fragment			
RstA-mut1-xbal-F	GCTCTAGAGGCTATAATCCAAACAATGG	1968	This study
RstA-mut2-smaI-R	GCCCCGGGTTAACTAAAGAATTACGTGG		
3-4 fragment			
RstA-mut3-smaI-F	GCCCCGGGTTACCGTCAGAATGTGTTTG	1957	This study
RstA-mut4-apaI-R	GCGGGCCCCCTTTATCCATATCAATTCC		
**PhlyC (*$hlyA_{ch}$*) screening**			
phlyC-5’	AATGTTTCTTTCCGTTGGGC	353	Terceti *et al.*, 2018
phlyC-3’	CCGGAGTTCCACCAGTAAAT		
**PhlyP (*$hlyA_{pl}$*) screening**			
PhlyP-5’	GCTATAAATGAATAAGAAAA	767	Terceti *et al.*, 2016
PhlyP-3’	TTGAAGCTAACTCAAAAA		
**Dly (*dly*) screening**			
Dly-5’	CTCCTATGGGACATGAATGG	549	Terceti *et al.*, 2016
Dly-3’	TGCTCTAGGCTAAATGAATC		
**VDA_000112 screening**			
VDA_000112 int -F	CATCAGATCTCAATGGTGCC	350	This study
VDA_000112 int -R	TCTTTAATAGGGTCTACGTA		
**VDA_002799 screening**			
VDA_002799 int-F	TCCATTTCGTTCTTGTTCGA	1500	Terceti *et al.*, 2018
VDA_002799 int-R	GATTTTTCAACGGACGATAT		
**A0J47_07530 screening**			
A0J47_07530 int-F	CATGGAGCCCCTAATGAACC	340	This study
A0J47_07530 int-R	AGTAAAATCAGCCGTTTGTT		
**VDA_000358 screening**			
VDA_000358 int -F	TGTCAAAGCATGGCTAGTTG	410	This study
VDA_000358 int -R	AGTCGAGAAAACAATCGCAT		
**RstAB system (*rstAB*) screening**			
Kinase int - F	TCAGCTTCATAATCTTCTAG	236	Terceti *et al.*, 2018
Kinase int - R	AATAAGATTGTGAGTCTACG		

Supplementary Table S2. Homology and function of the *Photobacterium damselae* subsp. *damselae* proteins identified in this study using blastP.

		Homologue Matches			
**Protein ID**	**Predicted Function**	**Accesion no.**	**Species**	**Identities**	**E-value**
**VDA_002460** (T2SS dependent)	Hypothetical protein	WP_005300136.1	*P. damselae* subsp. *damselae*	100%	0.0
	Lipoprotein putative	EHN69669.1	*Aliivibrio fischeri*	26%	6e-48
	Lipoprotein putative	SGY87728.1	*Moritella viscosa*	29%	2e-38
	Lipoprotein putative	AUL94513.1	*Vibrio vulnificus*	27%	3e-38
**VDA_000694** (T2SS dependent)	Sialidase	WP_005305077.1	*P. damselae* subsp. *damselae*	100%	0.0
	Sialidase	WP_095465352.1	*Vibrio cholerae*	53%	0.0
	Sialidase	WP_067416827.1	*Enterovibrio coralii*	66%	0.0
	Sialidase	WP_078753142.1	*Enterovibrio nigricans*	64%	0.0
	Sialidase	WP_107273569.1	*Photobacterium phosphoreum*	65%	0.0
	Sialidase	WP_107210839.1	*Photobacterium kishitanii*	63%	0.0
	Sialidase	WP_080159127.1	*Photobacterium piscicola*	61%	0.0
**A0J47_15850** (T2SS dependent)	Hypothetical protein	WP_068946047.1	*P. damselae* subsp. *damselae*	100%	0.0
	Hypothetical protein	WP_086775603.1	*Vibrio coralliirubri*	61%	6e-163
	Serine protease	WP_063437776.1	*Enterobacter cloacae*	40%	1e-107
	Peptidase S8	WP_012441023.1	*Erwinia tasmaniensis*	42%	5e-107
	Serine protease	WP_088205268.1	*Enterobacter bugandensis*	41%	1e-105
	Protease	CBJ46888.1	*Erwinia amylovora*	41%	4e-102
	Serine protease	WP_047054006.1	*Enterobacter hormaechei*	41%	5e-97
	Peptidase S8	WP_012147543.1	*Serratia proteamaculans*	39%	6e-98
**VDA_002799** (T2SS and RstAB dependent)	twin-arginine translocation pathway signal	WP_005301039.1	*P. damselae* subsp. *damselae*	100%	0.0
	twin-arginine translocation pathway signal	WP_044176547.1	*P. damselae* subsp. *piscicida*	99%	0.0
	twin-arginine translocation pathway signal	KJG31257.1	*Photobacterium angustum*	53%	0.0
	twin-arginine translocation pathway signal	WP_048246392.1	*Laetiporus sulphureus*	35%	3e-84
	twin-arginine translocation pathway signal	WP_059958603.1	*Burkholderia cepacia*	34%	2e-82
**A0J47_09785** (T2SS dependent)	Hypothetical protein	ODA21114.1	*P. damselae* subsp. *damselae*	100%	0.0
	trypsin family protein	WP_044175593.1	*P. damselae* subsp. *piscicida*	30%	2e-23
	peptidase S1 and S6, chymotrypsin/Hap	OAA35492.1	*Cordyceps brongniartii*	60%	3e-103
	Metalloprotease	PMB63986.1	*Beauveria bassiana*	58%	1e-99
	Hemolysin	WP_011517597.1	*Cupriavidus metallidurans*	53%	6e-96
	Hemolysin	WP_084624908.1	*Xanthomonas cassavae*	55%	2e-94

Supplementary Table S2. (cont.) Homology and function of the *Photobacterium damselae* subsp. *damselae* proteins identified in this study using blastP.

Protein ID	Predicted Function	Homologue Matches			
		**Accesion no.**	**Species**	**Identities**	**E-value**
**VDA_000112** (T2SS and RstAB dependent)	Hypothetical protein	WP_005306883.1	*P. damselae* subsp. *damselae*	100%	0.0
	Hypothetical protein	WP_023602899.1	*Aliivibrio logei*	40%	3e-57
	Hypothetical protein	WP_012552220.1	*Aliivibrio salmonicida*	39%	3e-59
	Hypothetical protein	WP_005431384.1	*Vibrio campbellii*	39%	4e-58
	Hypothetical protein	WP_075478204.1	*Moritella viscosa*	41%	2e-56
	Lipase chaperone	WP_061066385.1	*Vibrio harveyi*	39%	5e-56
	sugar-binding protein	WP_110077592.1	*Clostridium perfringens*	35%	1e-37
**VDA_000358** (T2SS and RstAB dependent)	Hypothetical protein	SPY44430.1	*P. damselae* subsp. *damselae*	100%	0.0
	Hypothetical protein	WP_068968745.1	*P. damselae* subsp. *piscicida*	96%	2e-168
	Hypothetical protein	WP_107282639.1	*Photobacterium lipolyticum*	30%	3e-20
	Hypothetical protein	WP_107253499.1	*Photobacterium indicum*	32%	4e-17
	Hypothetical protein	WP_006231910.1	*Photobacterium profundum*	30%	2e-15
**VDA_000966** (T2SS dependent)	Hypothetical protein	WP_005305856.1	*P. damselae* subsp. *damselae*	100%	3e-135
	Porin family protein	WP_115060987.1	*Photobacterium damselae*	96%	2e-133
	Hypothetical protein	WP_044178211.1	*P. damselae* subsp. *piscicida*	96%	2e-126
	Porin family protein	WP_001960583.1	*Vibrio cholerae*	40%	4e-32
	Porin family protein	WP_104970264.1	*Vibrio diabolicus*	48%	4e-48
	Porin family protein	WP_025535933.1	*Vibrio parahaemolyticus*	46%	1e-46
**VDA_000738** (T2SS dependent)	competence ComEA helix-hairpin-helix repeat	WP_005305210.1	*P. damselae* subsp. *damselae*	100%	1e-105
	competence ComEA helix-hairpin-helix repeat	WP_044179754.1	*P. damselae* subsp. *piscicida*	99%	9e-109
	competence ComEA helix-hairpin-helix repeat	KFE32301.1	*Vibrio cholerae*	57%	2e-15
	competence ComEA helix-hairpin-helix repeat	WP_105062336.1	*Photobacterium angustum*	64%	4e-58
	competence ComEA helix-hairpin-helix repeat	WP_107234348.1	*Photobacterium leiognathi*	60%	3e-54
	competence ComEA helix-hairpin-helix repeat	WP_006229811.1	*Photobacterium profundum*	57%	1e-50
	competence ComEA helix-hairpin-helix repeat	WP_107252572.1	*Photobacterium indicum*	56%	7e-50
**AJ047_07530** (T2SS and RstAB dependent)	Hypothetical protein	WP_036764371.1	*P. damselae* subsp. *damselae*	100%	0.0
	Hypothetical protein	WP_086957603.1	*P. damselae* subsp. *piscicida*	96%	7e-90
	Hypothetical protein	WP_009601554.1	*Vibrio caribbeanicus*	32%	1e-09
	Hypothetical protein	WP_071102108.1	*Moorea producens*	34%	1e-04
	Hypothetical protein	WP_036755189.1	*Photobacterium galatheae*	31%	2e-05

Supplementary Table S3. Analysis of the proteins identified in this study by Pfam 31.0.

Acession ID	Family/Domain	Description	Clan	Envelope		Alignment		Bit score	E-value
				start	end	start	end		
VDA_002460 *	-	-	-	-	-	-	-	-	-
VDA_000694	Sial-lect-inser (PF09264.9)	*Vibrio cholerae* sialidase, lectin insertion	CL0004	67	233	82	214	36.7	8e-09
	Sial-lect-inser (PF09264.9)	*Vibrio cholerae* sialidase, lectin insertion	CL0004	345	542	346	542	105.6	2e-30
	BNR_2 (PF13088.5)	BNR repeat-like domain	CL0434	493	751	546	746	53.2	4e-14
A0J47_15850	Peptidase_S8 (PF00082.21)	Subtilase family	N/a	71	458	207	442	53.2	4e-14
VDA_002799	Endotoxin_N (PF03945.13)	delta endotoxin, N-terminal domain	N/a	65	273	68	273	73.5	8e-20
A0J47_09785	Trypsin (PF00089.25)	Trypsin	CL0124	50	273	63	273	31.5	4e-07
VDA_000112*	-	-	-	-	-	-	-	-	-
VDA_000358*	-	-	-	-	-	-	-	-	-
VDA_000966	OMP_b-brl (PF13505.5)	Outer membrane protein beta-barrel domain	CL0193	9	190	13	190	49.2	1e-13
VDA_000738	HHH_3 (PF12836.6)	Helix-hairpin-helix motif	CL0198	95	160	96	159	73.6	1e-20
AJ047_07530*	-	-	-	-	-	-	-	-	-

* No matches found

Supplementary Figure S1. Phylogenetic trees representing the relationship between RstA (top) and RstB (bottom) proteins of *Photobacterium damselae* subsp. *damselae* RM-71, and homologous proteins from related bacteria.

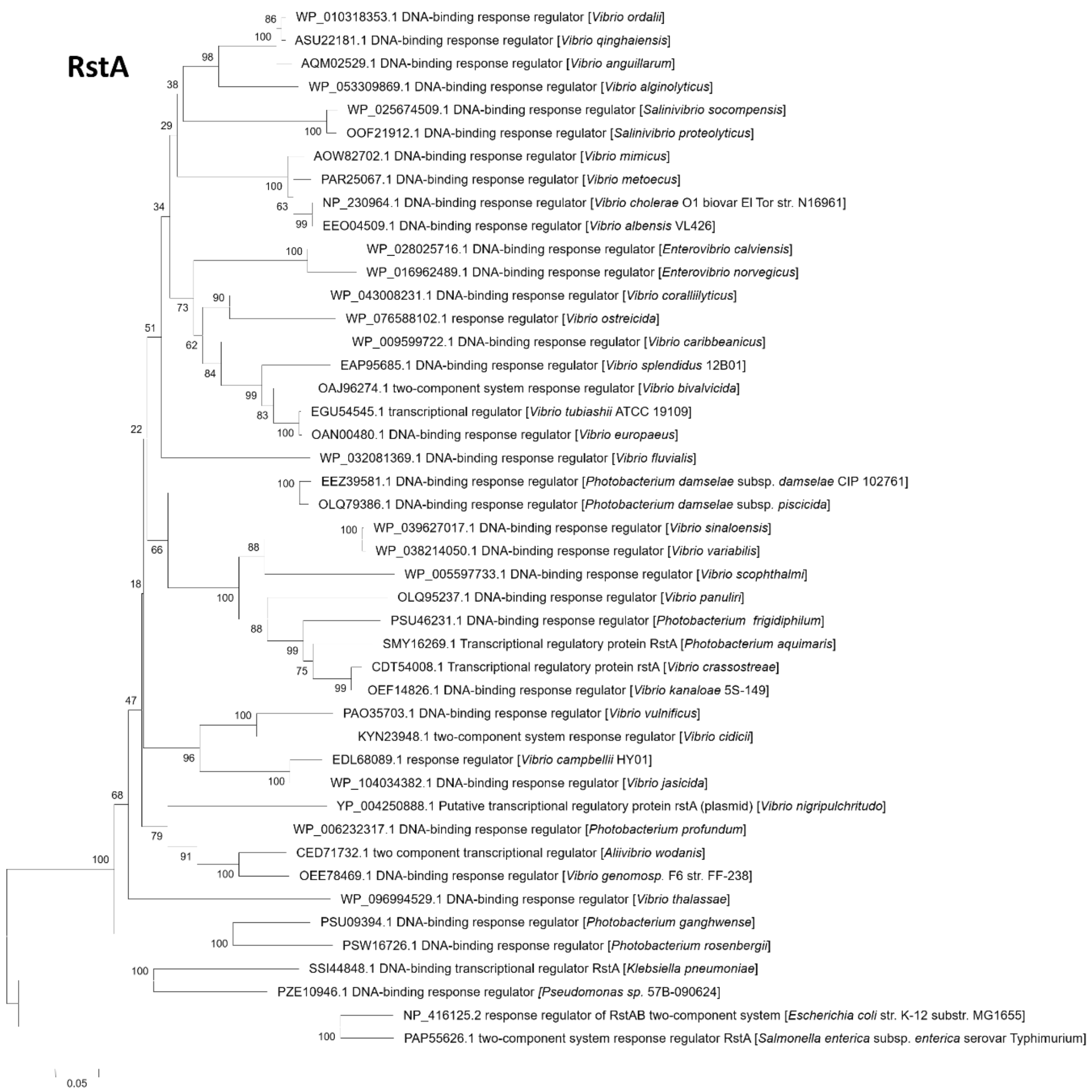

RstB

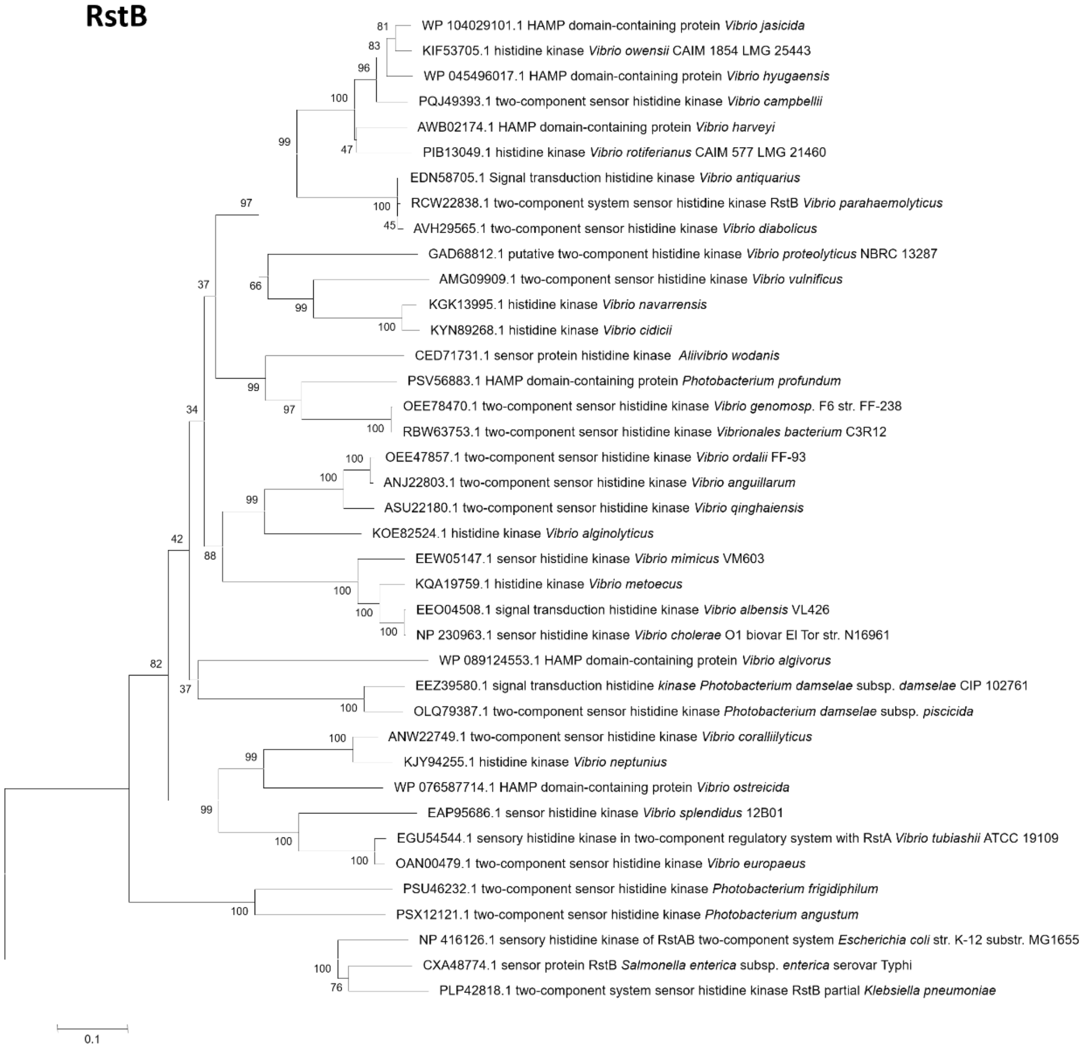

WP 104029101.1 HAMP domain-containing protein Vibrio jasicida
KIF53705.1 histidine kinase Vibrio owensii CAIM 1854 LMG 25443
WP 045496017.1 HAMP domain-containing protein Vibrio hyugaensis
PQJ49393.1 two-component sensor histidine kinase Vibrio campbellii
AWB02174.1 HAMP domain-containing protein Vibrio harveyi
PIB13049.1 histidine kinase Vibrio rotiferianus CAIM 577 LMG 21460
EDN58705.1 Signal transduction histidine kinase Vibrio antiquarius
RCW22838.1 two-component system sensor histidine kinase RstB Vibrio parahaemolyticus
AVH29565.1 two-component sensor histidine kinase Vibrio diabolicus
GAD68812.1 putative two-component histidine kinase Vibrio proteolyticus NBRC 13287
AMG09909.1 two-component sensor histidine kinase Vibrio vulnificus
KGK13995.1 histidine kinase Vibrio navarrensis
KYN89268.1 histidine kinase Vibrio cidicii
CED71731.1 sensor protein histidine kinase Aliivibrio wodanis
PSV56883.1 HAMP domain-containing protein Photobacterium profundum
OEE78470.1 two-component sensor histidine kinase Vibrio genomosp. F6 str. FF-238
RBW63753.1 two-component sensor histidine kinase Vibrionales bacterium C3R12
OEE47857.1 two-component sensor histidine kinase Vibrio ordalii FF-93
ANJ22803.1 two-component sensor histidine kinase Vibrio anguillarum
ASU22180.1 two-component sensor histidine kinase Vibrio qinghaiensis
KOE82524.1 histidine kinase Vibrio alginolyticus
EEW05147.1 sensor histidine kinase Vibrio mimicus VM603
KQA19759.1 histidine kinase Vibrio metoecus
EEO04508.1 signal transduction histidine kinase Vibrio albensis VL426
NP 230963.1 sensor histidine kinase Vibrio cholerae O1 biovar El Tor str. N16961
WP 089124553.1 HAMP domain-containing protein Vibrio algivorus
EEZ39580.1 signal transduction histidine kinase Photobacterium damselae subsp. damselae CIP 102761
OLQ79387.1 two-component sensor histidine kinase Photobacterium damselae subsp. piscicida
ANW22749.1 two-component sensor histidine kinase Vibrio coralliilyticus
KJY94255.1 histidine kinase Vibrio neptunius
WP 076587714.1 HAMP domain-containing protein Vibrio ostreicida
EAP95686.1 sensor histidine kinase Vibrio splendidus 12B01
EGU54544.1 sensory histidine kinase in two-component regulatory system with RstA Vibrio tubiashii ATCC 19109
OAN00479.1 two-component sensor histidine kinase Vibrio europaeus
PSU46232.1 two-component sensor histidine kinase Photobacterium frigidiphilum
PSX12121.1 two-component sensor histidine kinase Photobacterium angustum
NP 416126.1 sensory histidine kinase of RstAB two-component system Escherichia coli str. K-12 substr. MG1655
CXA48774.1 sensor protein RstB Salmonella enterica subsp. enterica serovar Typhi
PLP42818.1 two-component system sensor histidine kinase RstB partial Klebsiella pneumoniae
0.1
